# The association of C-reactive protein with left ventricular systolic function and left ventricular myocardial fibrosis

**DOI:** 10.1186/1532-429X-11-S1-P45

**Published:** 2009-01-28

**Authors:** Mohammed Ahmed, Ijaz Ahmad, Munawar Hayat, Jasmin Ullah, Nadeem Asad, Joshua A Socolow, Igor Klem, Matt Briggs, Terrence Sacchi, John F Heitner

**Affiliations:** 1 New York Methodist Hospital, Brooklyn, NY USA; 2 Duke University Medical Center, Durham, NC USA

**Keywords:** Congestive Heart Failure, Leave Ventricular Ejection Fraction, Peripheral Vascular Disease, Myocardial Fibrosis, Ischemic Cardiomyopathy

## Background

C-reactive Protein (CRP) is a serologic marker that has been shown to predict adverse cardiovascular outcomes. In addition, CRP has been shown to be elevated in patients with congestive heart failure (CHF).

## Objective

To evaluate if CRP is associated with the severity of left ventricular dysfunction, independent of the degree of myocardial fibrosis.

## Methods

We prospectively evaluated 46 outpatients referred to our cardiac MRI (CMR) center for the evaluation of ischemic cardiomyopathy (LVEF < 45%). Patients were excluded if they were discharged from the hospital within 7 days of the CMR study or were in acute CHF by clinical exam on the day of the study. Blood samples were drawn for the measurement of CRP on the day of the CMR. All patients had left ventricular function and amount of scar tissue calculated by computer analysis after ROI curves were performed.

## Results

The average age of the patients was 67.7 years (36 males and 10 females). The prevalence of the following risk factors was as follows: hyperlipidemia (80%), hypertension (77%), diabetes (50%), tobacco (14%), renal disease (11%), and peripheral vascular disease (7%). The mean left ventricular ejection fraction (LVEF) was 36.7%. The mean left ventricular myocardial fibrosis was 16 g. Separate regression models were applied for CRP predicting left ventricular myocardial fibrosis and LVEF. The log CRP was associated with both left ventricular myocardial fibrosis (p = 0.05) and LVEF (p = 0.03). In addition, the log CRP was associated with a low ejection fraction, independent of the amount of left ventricular myocardial fibrosis (p = 0.03).

## Conclusion

CRP is an independent predictor of left ventricular ejection fraction in patients with ischemic cardiomyopathy.

